# Support Vector Machine Analysis of Functional Magnetic Resonance Imaging of Interoception Does Not Reliably Predict Individual Outcomes of Cognitive Behavioral Therapy in Panic Disorder with Agoraphobia

**DOI:** 10.3389/fpsyt.2017.00099

**Published:** 2017-06-09

**Authors:** Benedikt Sundermann, Jens Bode, Ulrike Lueken, Dorte Westphal, Alexander L. Gerlach, Benjamin Straube, Hans-Ulrich Wittchen, Andreas Ströhle, André Wittmann, Carsten Konrad, Tilo Kircher, Volker Arolt, Bettina Pfleiderer

**Affiliations:** ^1^Department of Clinical Radiology, University Hospital Münster, Münster, Germany; ^2^Department of Psychology, Institute of Clinical Psychology and Psychotherapy, Technische Universität Dresden, Dresden, Germany; ^3^Center for Mental Health, Department of Psychiatry, Psychosomatics, and Psychotherapy, University Hospital Würzburg, Würzburg, Germany; ^4^Klinische Psychologie und Psychotherapie, Universität zu Köln, Cologne, Germany; ^5^Department of Psychiatry and Psychotherapy, Philipps University of Marburg, Marburg, Germany; ^6^Department of Psychiatry and Psychotherapy, Charité – University Medicine Berlin, Berlin, Germany; ^7^Department of Psychiatry and Psychotherapy, Agaplesion Diakonieklinikum Rotenburg, Rotenburg, Germany; ^8^Department of Psychiatry and Psychotherapy, University Hospital Münster, Münster, Germany; ^9^Otto Creutzfeldt Center for Cognitive and Behavioral Neuroscience, University of Münster, Münster, Germany

**Keywords:** panic disorder, agoraphobia, cognitive behavioral therapy, interoception, functional magnetic resonance imaging, diagnostic classification, machine learning, support vector machines

## Abstract

**Background:**

The approach to apply multivariate pattern analyses based on neuro imaging data for outcome prediction holds out the prospect to improve therapeutic decisions in mental disorders. Patients suffering from panic disorder with agoraphobia (PD/AG) often exhibit an increased perception of bodily sensations. The purpose of this investigation was to assess whether multivariate classification applied to a functional magnetic resonance imaging (fMRI) interoception paradigm can predict individual responses to cognitive behavioral therapy (CBT) in PD/AG.

**Methods:**

This analysis is based on pretreatment fMRI data during an interoceptive challenge from a multicenter trial of the German PANIC-NET. Patients with DSM-IV PD/AG were dichotomized as responders (*n* = 30) or non-responders (*n* = 29) based on the primary outcome (Hamilton Anxiety Scale Reduction ≥50%) after 6 weeks of CBT (2 h/week). fMRI parametric maps were used as features for response classification with linear support vector machines (SVM) with or without automated feature selection. Predictive accuracies were assessed using cross validation and permutation testing. The influence of methodological parameters and the predictive ability for specific interoception-related symptom reduction were further evaluated.

**Results:**

SVM did not reach sufficient overall predictive accuracies (38.0–54.2%) for anxiety reduction in the primary outcome. In the exploratory analyses, better accuracies (66.7%) were achieved for predicting interoception-specific symptom relief as an alternative outcome domain. Subtle information regarding this alternative response criterion but not the primary outcome was revealed by *post hoc* univariate comparisons.

**Conclusion:**

In contrast to reports on other neurofunctional probes, SVM based on an interoception paradigm was not able to reliably predict individual response to CBT. Results speak against the clinical applicability of this technique.

## Introduction

Applying multivariate pattern analysis (MVPA) techniques from the field of machine learning to functional magnetic resonance imaging (fMRI) data has been proposed as a strategy to develop diagnostic or predictive tools for mental disorders. MVPA integrates potentially discriminative information from multiple brain locations, states, or imaging modalities instead of analyzing them in insolation. MVPA techniques are applied to learn decision rules (classifiers) based on labeled training data. These rules are subsequently applied to diagnostically label previously unseen data (1–8). Particularly, support vector machines (SVM) have been used to extract meaningful information from noisy and high-dimensional fMRI data (5, 6). Predicting individual therapeutic outcomes in psychiatric patients (including those with anxiety disorders) is an emergent focus of these methodological efforts (9–15). Responses to pharmacotherapy and to psychotherapy have been proposed to be mediated by neurobiological factors (12, 14, 16–21). Therefore, predictive neuroimaging biomarkers are of particular scientific interest as candidate tools to guide clinical treatment decision in individual psychiatric patients (9, 14, 22).

Cognitive behavioral therapy (CBT) alone or in combination with pharmacotherapy is the first-line treatment of choice in patients suffering from panic disorder with agoraphobia (PD/AG) (23–29). While there is a clinically significant proportion of non-responders (26, 28, 30), there is only incipient but rapidly increasing knowledge of moderators and predictors of CBT response (12, 31, 32). Neural correlates of CBT in PD/AG have been investigated in fMRI group comparison studies suggesting a role of altered neural activity in networks regulating negative emotions as well as fear conditioning and extinction (12, 18, 21, 33–36). Recently, first reports of successful applications of MVPA to predict individual CBT outcomes in PD/AG based on fMRI emerged: Hahn et al. reported an overall diagnostic accuracy of up to 82% using Gaussian process classifiers (GPCs) in a meta-learning scheme to train models based on task fMRI data from a fear condition paradigm in an overlapping sample (11). Ball et al. utilized random forest classification based on an emotion regulation task. They reached accuracies of 79% in a mixed sample of PD and generalized anxiety disorder and 85% in the PD subsample (37).

An intensified and abnormal internal focus of attention to bodily sensations (interoception) is a characteristic feature observed in PD/AG (38, 39). This comprises increased self-report of bodily symptoms (particularly cardiac) as well as their dysfunctional cognitive appraisal including catastrophizing (39). Interoception is therefore assumed to be an important determinant of maintenance of PD/AG (28, 38, 39). It is thus specifically addressed by CBT *via* interoceptive exposure (28). Interoception can be effectively studied by fMRI and is associated with activity in a widespread cerebral network overlapping with established fear circuitry models (39–42).

The purpose of this investigation was to assess whether fMRI based on an interoception task acquired at multiple sites combined with SVM, a well-established MVPA technique, can predict CBT response of individual patients with PD/AG. Beyond that, we aimed at further exploring the influence of methodological decisions and the predictive ability for specific interoception-related symptom reduction. We additionally performed univariate group analyses comparing responders and non-responders to assess feature set information content and to evaluate the general suitability of the paradigm to detect neural processes related to therapy response.

## Materials and Methods

### Subjects

This investigation is based on fMRI data in a subset of patients of the multicenter, randomized-controlled trial “Mechanism of action in CBT” (MAC) (43) within the framework of the German research network PANIC-NET (44). Primary goal of the MAC trial is to identify mechanisms through which CBT achieves its beneficial effects as well as mediators and moderators of response. It involves the acquisition of a broad spectrum of clinical, behavioral, physiological, experimental, and genetic data. Written informed consent was obtained from all participants in accordance with the Declaration of Helsinki. The randomized clinical trial (isrctn.org identifier: ISRCTN80046034) was approved by the ethics committee of the Medical Faculty of the Technische Universität Dresden (agreement EK 164082006). The neuroimaging components were approved by the ethics committee of the Medical Faculty of the RWTH Aachen University, Aachen (agreement EK 073/07) and at all local sites (43).

The overall MAC sample involved adult outpatients (*n* = 369) who met criteria for a current primary diagnosis of PD/AG (43) according to DSM-IV-TR (45). Only moderate exclusion criteria were adopted to allow for typical comorbidity seen in routine care. They comprised comorbid psychotic or bipolar I disorders, current substance dependence or abuse, a current suicidal intent, borderline personality disorder, ongoing psychotherapeutic or psychopharmacological treatment as well as procedure-specific contraindications. Psychometric assessments in participants of the fMRI substudy included the Hamilton Scale for Anxiety (HAM-A) (46, 47), Beck Depression Inventory (BDI-II) (48), Anxiety Sensitivity Index (49), Clinical Global Impression (50), PD/AG Scale (51), trail-making task (52), and digit span task from the German Wechsler Adult Intelligence Scale—Revision IV (53). Patients received manualized exposure-based CBT encompassing 12× 100 min treatment sessions (two subgroups either with or without therapist-guided exposure) or were allocated to a wait-list control group (data not used in this analysis). Please see the MAC methods paper for full details on patient recruitment, treatment, and data collection (43). A subgroup of patients (*n* = 89) were invited to participate in the fMRI substudy (33). This analysis is based on a subsample (*n* = 59) representing all PD/AG patients who completed CBT (including the assessment of clinical outcomes) as well as an interoception fMRI task (41) at baseline and fMRI data quality assessment (see Figure S1 in Supplementary Material for a flowchart of patient selection). Analogous to Hahn et al. (11) a reduction in HAM-A scores ≥50% (primary outcome) from baseline to posttreatment assessment was used as a standard criterion for treatment response (43). Data from responders (*n* = 30) and non-responders (*n* = 29) were analyzed here. Demographic and clinical details of responders and non-responders are presented in Table 1. Statistical assessment of clinical and demographical data was accomplished using IBM SPSS Statistics (version 22, IBM, Armonk, NY, USA, RRID:SCR_002865).

**Table 1:** Basic characteristics of responders and non-responders to cognitive behavioral therapy in the main analysis (primary outcome, responder-threshold: 50% HAM-A reduction compared to pretreatment baseline).

		Responders	Non-responders	Test statistic	df	*p*
Number		30	29			

Sex	Female	18 (60.0%)	19 (65.5%)	χ^2^ = 0.192	1	0.661^c^
	Male	12 (40.0%)	10 (34.5%)			

Age (years)		36.8 ± 12.2	37.3 ± 10.1	*t* = −0.198	57	0.844^a^

Education	Lower secondary	13 (43.3%)	18 (62.1%)	χ^2^ = 2.990	2	0.212^c^
	Higher secondary	11 (36.7%)	9 (31.0%)			
	University	6 (20.0%)	2 (6.9%)			

Site (*n*)	Aachen	0 (0.0%)	2 (6.9%)	χ^2^ = 4.140	3	0.247^c^
	Berlin	11 (36.7%)	7 (24.1%)			
	Dresden	10 (33.3%)	14 (48.3%)			
	Münster	9 (30.0%)	6 (20.7%)			

CBT arm	Therapist-guided	14 (46.7%)	19 (65.5%)	χ^2^ = 2.126	1	0.192^c^
	Non-guided	16 (53.3%)	10 (34.5%)			

Randomized first fMRI condition (*n*)	Interoception	17 (56.7%)	11 (37.9%)	χ^2^ = 2.076	1	0.150^c^
	Exteroception	13 (43.3%)	18 (62.1%)			

HAM-A	Before CBT	24.0 ± 5.5	25.1 ± 5.3	*t* = −0.784	57	0.436^a^
	After CBT	7.9 ± 3.3	18.1 ± 5.2	*t* = −8.996	46.7	<0.001^a^^,^*

BDI-II	Before CBT	15.9 ± 9.9	17.0 ± 7.9	*t* = −0.469	57	0.641^a^
	After CBT	6.5 ± 5.3	12.9 ± 8.8	*t* = −3.361	45.4	0.002^a^^,^*

ASI	Before CBT	30.9 ± 9.7	31.0 ± 12.1	*t* = −0.025	57	0.980^a^
	After CBT	12.9 ± 6.8	18.8 ± 10.4	*t* = −2.580	57	0.012^a^^,^*

CGI	Panic symptoms	5 (4–7)	5 (4–7)			0.570^b^
	Anxiety	3 (1–6)	4 (3–5)			0.009^b^^,^*

PAS		21.0 ± 8.2	29.6 ± 6.2	*t* = −4.553	57	<0.001^a^^,^*

TMT (s)	A	26.3 ± 9.5	27.1 ± 8.3	*t* = −0.337	57	0.737^a^
	B	59.6 ± 20.1	58.5 ± 17.7	*t* = 0.210	57	0.834^a^

Digit span task	Total	15.1 ± 2.8	14.2 ± 3.1	*t* = 1.152	57	0.254^a^

Comorbid depression (*n*)^d^	Before CBT	10 (33.3%)	9 (31.0%)	χ^2^ = 0.036	1	0.850^c^
	After CBT	1 (3.7%)	6 (20.7%)	χ^2^ = 4.248	1	0.039^c^^,^*

All test results without further specification were obtained at the first visit at base line assessment of CBT and represent “number (percentage),” “mean ± SD” or “median (range).”

*denotes statistical significance (*p* < 0.05).

*^a^t-test*.

*^b^Mann–Whitney U-test*.

*^c^χ^2^-test*.

*^d^based on BDI-II scores*.

*CBT, cognitive behavioral therapy; HAM-A, Hamilton Scale for Anxiety; BDI-II, Beck Depression Inventory II; ASI, Anxiety Sensitivity Index; CGI, Clinical Global Impression; PAS, PD/AG Scale; TMT, trail-making task*.

A secondary response criterion was available in a subgroup of 54 out of 59 patients (see Multivariate Classification on exploratory analyses): this was based on the assessment of the intensity of bodily symptoms (SI) and experienced anxiety (EA) during two sessions of “interoceptive exposure” to bodily sensations during CBT (43, 54). Self-report data on SI and EA were documented on a scale from 0 to 10 before (fourth CBT session) and after (fifth CBT session) an interoceptive exercise (IE) involving repeated self-guided exposure at home. The interoceptive exposure involved a wide range of bodily stimuli. To calculate the summary “interoceptive” score used here, the summary measures of the three stimuli with largest effect sizes: breathing through a straw, rotating around the longitudinal body axis, and hyperventilation were chosen (54). Absolute between-session differences of SI and EA scores were averaged over these three stimuli to obtain cumulated measures of SI or EA reduction in individual patients. Patients with a cumulated SI and EA reduction above or below the group mean were classified as responders and non-responders, respectively. This alternative response criterion resulted in a further subgrouping of IE responders (*n* = 26) and IE non-responders (*n* = 28). Interoception-based response was not associated with overall response based on 50% HAM-A reduction (χ^2^ = 0.297, *p* = 0.586). See Table S1 in Supplementary Material for clinical and demographical characteristics of IE responders and non-responders.

### fMRI Data Acquisition and First-Level Analyses

Data acquisition was accomplished at four imaging centers using T2*-weighted gradient echo echo-planar imaging (225 volumes, 36 slices, matrix 64 × 64, field of view 210 mm, reconstructed as 3.6 mm × 3.6 mm × 3.6 mm voxels, echo time 35 ms, repetition time 3,000 ms, and flip angle 90°) at 3 T (Aachen and Münster: Achieva, Philips, Best, Netherlands; Berlin: GE Healthcare, Little Chalfont, UK; Dresden: Magnetom Trio, Siemens, Erlangen, Denmark).

During the fMRI scan, participants performed a mental tracking paradigm (41) adapted from the so-called “Schandry task” (55). The paradigm examines the effects of focusing one’s attention internally (interoception) vs. externally (exteroception) using a block design with four blocks. External stimuli were identical in both conditions: hard to hear clicking sounds (*n* = 104 per block): during exteroception, participants were instructed to silently count the clicking sounds and to subsequently report the number of clicks. During interoception, participants were instructed to silently count their own heartbeats and to report the number of heartbeats counted in a particular interval. Subjects were randomized to either start with an interoception or exteroception block (see Table 1). In total, two blocks of interoception (I1, I2) and two blocks of exteroception (E1, E2) were presented. This paradigm had been validated previously in anxiety sensitive females (41).

Preprocessing and first-level analyses were conducted with SPM5^1^ (RRID:SCR_007037). Images were realigned, normalized and resliced (voxel size 2 mm × 2 mm × 2 mm), and smoothed with a Gaussian kernel (full width at half maximum: 8 mm). Movement correction parameters were used as regressors in the first-level model. Data were filtered with a high-pass filter (cutoff period of 128 s). The two blocks of interoception and the two blocks of exteroception, respectively, were added to build one condition (I = I1 + I2; E = E1 + E2) (41).

### Multivariate Classification

Maps representing either the simple contrast “interoception” (I) (i.e., the respective beta-map) or the differential contrast “interoception > exteroception” (I > E) in individual subjects were used as features for subsequent multivariate classification and *post hoc* univariate group comparisons.

#### General Approach and Hypothesis Tests

Modeling and validation were implemented using the Machine learning Application for NeuroImaging Analyses (MANIA, version 2.5) (56). Diagnostic performance was assessed using leave-one-out cross-validation (57). The statistical significance of inferentially tested methods was estimated using permutation testing (58) with 100 permutations.

Support vector machine models tested here were based on soft-margin support vector classification (C-SVC) from LIBSVM (59). In SVM models, a hyperplane is defined in order to distinguish between responders and non-responders. Models are optimized using a kernel by maximizing the margin of separation between groups based on the datasets closest to the hyperplane. Model parameters can be chosen to adjust model complexity. In this particular case, the penalty-term C adjusts the models’ tolerance for misclassifications in the training dataset (57, 60–62).

**Figure 1:**
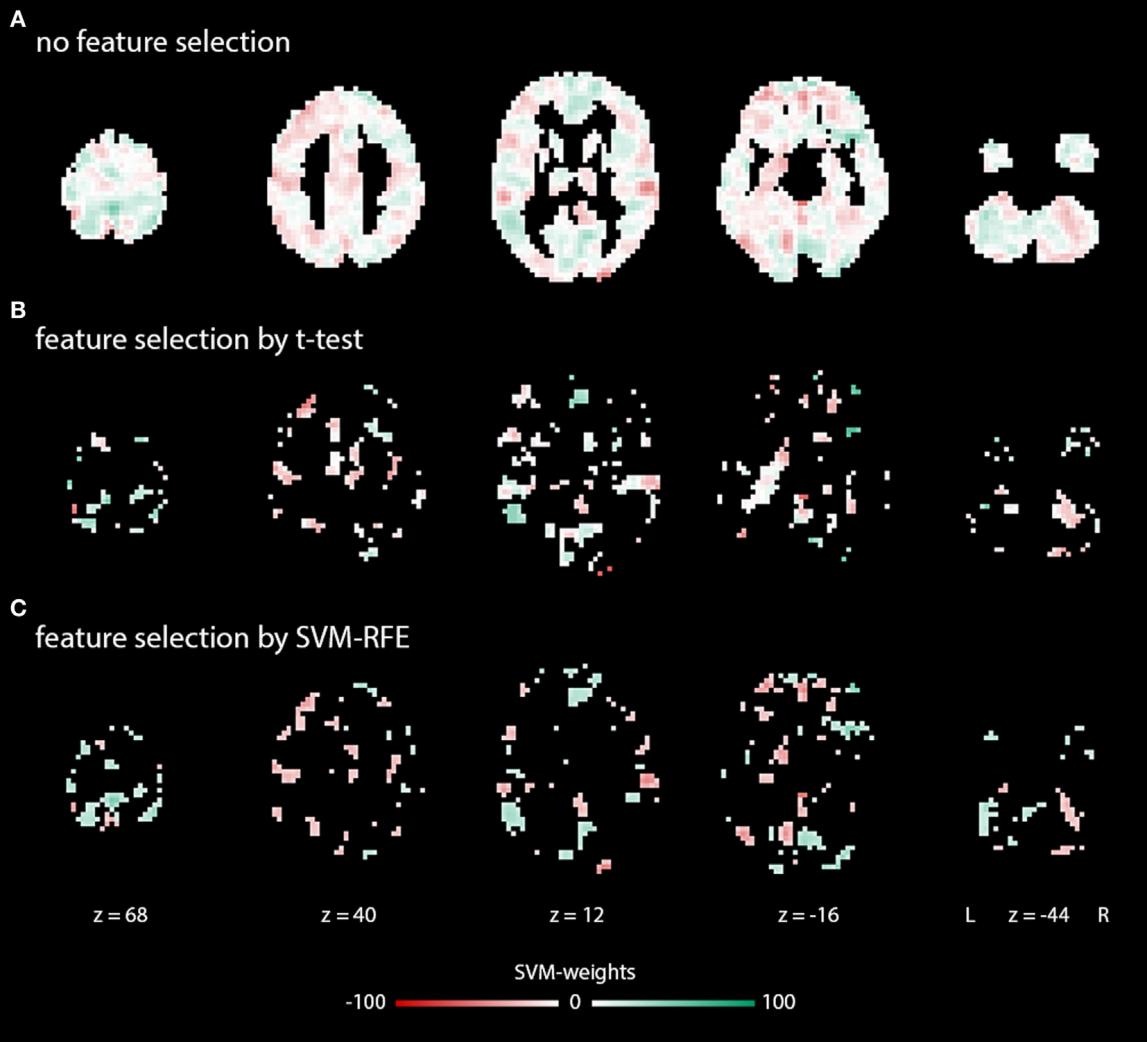
Exemplary weight maps illustrating typical feature sets in the main analysis: **(A)** without feature selection (FS), **(B)** with FS by a *t*-test-based filter, **(C)** with FS by SVM-RFE. Illustrations were created using Mango (http://ric.uthscsa.edu/mango/).

Support vector machines can be combined with different methods for dimensionality reduction and feature selection (FS) with the aim to improve diagnostic accuracies (57, 63). In this study, whole-brain datasets were masked to reduce dimensionality and preselect features (64). Additionally, models were tested either without any automated FS, with a simple filter (based on results of a two-sample *t*-test) or with recursive feature elimination using linear SVMs (SVM-RFE). SVM-RFE is an iterative procedure in which unimportant features are removed based on their SVM weights. Compared to the simple filters, SVM-RFE takes dependencies among features into account (65). Here, we used the greedy approach to SVM-RFE (56, 66). The effects of FS on an exemplary feature set are illustrated in Figure 1.

We performed hypothesis-based tests on six different models with standard methodological choices to test whether these models were able to identify individual responders and non-responders (50% HAM-A reduction) based on pretreatment fMRI: Contrast-maps (I, I > E) were downsampled to a voxel size of 4 mm × 4 mm × 4 mm using SPM representing the rounded original voxel size in order to limit feature set dimensionality by avoiding redundancies only introduced during image registration. The Automated Anatomical Labeling (AAL) atlas (67) resampled to an identical resolution was used as a mask to restrict analyses predominantly to voxels representing gray matter. For each of the two contrasts (I, I > E), we assessed modeling without further automated FS, with a *t*-test filter and with SVM-RFE. For models with FS, the number of features to be selected was *n* = 4,557 (20% of voxels within the AAL mask). We did not further restrict the number of features here to avoid models based on only one or few coherent brain region comprising multiple voxels. For all six linear C-SVC models, we chose an intermediate value of the penalty term (C = 1), the default setting in LIBSVM.

#### Further Exploratory Analyses

Further potentially influencing factors were tested separately in exploratory analyses to identify factors that may improve diagnostic performance: (i) to assess whether interoception-related symptom relief would be a better predictable therapy response by SVM based on this fMRI interoception paradigm, we used the results of the alternative response criterion taking into account the response to an interoceptive challenge (IE responders vs. IE non-responders) (54). This contrasts with the standard HAM-A-based response criterion which focusses on clinically relevant general anxiety reduction. (ii) Different methodological decisions (feature set preparation, classification algorithm, modeling parameters, and software): the cost parameter was varied systematically C = {0.01, 1, 100} for all analogous analyses with the main hypothesis tests and for all tests described hereafter. Another automated FS method was evaluated: selection by SVM weights. The influence of further gray matter masks was assessed: the cortical or subcortical Harvard-Oxford atlas (68–71) and a combined mask including the bilateral amygdala and parahippocampal gyrus as defined by Talairach labels (72, 73) in the WFU PickAtlas toolbox.^2^ The voxel size was kept either at its level after preprocessing (2 mm × 2 mm × 2 mm) or further increased to 6 mm × 6 mm × 6 mm in order to modify the feature set dimensionality. When different voxel resolutions were combined with FS, the number of features to be selected was adjusted to constantly reflect 20% of voxel within in the respective resampled mask. The Pattern Recognition for Neuroimaging Toolbox (PRoNTo, version 1.1, RRID:SCR_006908) (64) was used instead of MANIA. Both toolboxes rely on C-SVC from LIBSVM. However, there are some conceptual differences: while MANIA adopts the original linear C-SVC implementation from LIBSVM, PRoNTo uses precomputed linear kernels that are subsequently passed to LIBSVM. Both toolboxes are relatively new scientific software. We thus also wanted to exclude that simple data handling issues significantly influence MVPA results. Finally, we tested GPCs based on the Gaussian Processes for Machine Learning (74) implementation in MANIA (mean function: mean zero, covariance function: linear, likelihood function Likert). All analysis steps and parameters not explicitly mentioned here were identical with the inferentially tested models.

### *Post Hoc* Univariate Group Comparisons

In order to assess the information content of the fMRI data to interpret multivariate classification performance, we additionally conducted a conventional univariate whole-brain analysis of response effects after therapy in patients. These were carried out with SPM. The “full factorial” design option was used with the factor “response to therapy” (yes, no) as independent factor. Separate analyses of variance were performed for both, the simple contrast I and the differential contrast I > E for both response criteria (HAM-A reduction and interoception criterion). The main effect of response was assessed. Results were cluster-size corrected for multiple comparisons on the cluster level at *p* < 0.05. This was equivalent to *p* < 0.001 with a minimum cluster extent of *k* = 42 contiguous resampled voxels with our given scanning parameters as estimated based on a Monte Carlo simulation implemented in Matlab (75).

## Results

### Performance of Inferentially Tested Standard Approaches

Tested models did not yield significant diagnostic accuracies to identify individual responders and non-responders (50% HAM-A reduction) based on pretreatment fMRI with an interoception task. Overall accuracies ranged from 39.0 to 54.2% with sensitivities from 30.0 to 50.0% and specificities from 37.9 to 58.6%. See Table 2 for detailed results.

**Table 2:** Results of main classification approaches to predict general anxiety reduction after CBT (hypothesis tests).

GLM contrast	Feature selection (FS)	Accuracy (*p*)	Sensitivity (%)	Specificity (%)
I	–	39.0% (0.89)	36.7	41.4
I	*t*-Test filter	39.0% (0.91)	40.0	37.9
I	SVM-RFE	39.0% (0.91)	36.7	41.4

I > E	–	39.0% (0.89)	30.0	48.3
I > E	*t*-Test filter	54.2% (0.33)	50.0	58.6
I > E	SVM-RFE	42.4% (0.79)	40.0	44.8

*Models based on C-SVC (C = 1). FS to select 20% of voxels. Statistical significance assessed by permutation testing*.

*INT, interoception; EXT, exteroception; SVM-RFE, recursive feature elimination using support vector machines*.

### Exploratory Analyses

Further exploratory analyses aiming at methodological factors (feature set preparation, classification algorithm, modeling parameters, and software) did also not yield above-chance diagnostic performance. Overall accuracies ranged from 33.9 to 54.2% with sensitivities from 30.0 to 60.0% and specificities from 27.6 to 58.6%. An overview of models tested and detailed results are presented in Tables S2 and S3 in Supplementary Material.

Analyses with an alternative response criterion specifically aiming at a reduction of symptoms directly related to interoception (IE responders vs. IE non-responders) exhibited higher diagnostic accuracies compared with the standard response criterion. Overall accuracies ranged from 50.0 to 66.7% with sensitivities from 50.0 to 69.2% and specificities from 46.4 to 67.9%. See Table 3 for detailed results.

**Table 3:** Results of an exploratory analysis with an interoception-specific response criterion (prediction of a reduction of bodily symptoms and anxiety during an interoceptive task, IE-responders vs. IE-non-responders).

GLM contrast	Feature selection (FS)	Accuracy (*p*)	Sensitivity (%)	Specificity (%)
I	–	50.0% (0.48)	50.0	50.0
I	*t*-Test filter	63.0% (0.14)	69.2	57.1
I	SVM-RFE	48.2% (0.55)	50.0	46.4

I > E	–	57.4% (0.21)	57.7	57.1
I > E	*t*-Test filter	66.7% (0.02)	65.4	67.9
I > E	SVM-RFE	57.4% (0.15)	57.7	57.1

*p-Values are reported only in order to exemplify the relationship between the observed accuracies and the distribution of chance level accuracies, but do not reflect planned hypothesis tests*.

*Models based on C-SVC (C = 1). FS to select 20% of voxels. Statistical significance assessed by permutation testing*.

*INT, interoception; EXT, exteroception; SVM-RFE, recursive feature elimination using support vector machines*.

### *Post Hoc* Univariate Group Comparisons

Conventional univariate group comparisons revealed a main effect of therapy response only for the interoception-specific response criterion with the contrast I > E (Figure S2 in Supplementary Material). No significant effects were observed for the interoception-specific outcome with the simple contrast I or for the HAM-A based primary outcome (contrasts I and I > E).

## Discussion

Diagnostic modeling based on a pretreatment interoception task with standard fMRI and voxel-wise SVM including FS did not achieve significant accuracies to predict individual CBT response in a randomized, controlled multicenter study. Using these methodological choices, we could not reach diagnostic performances of alternative models with different fMRI tasks. Such models have been reported by Hahn et al. in a fear conditioning fMRI paradigm (Gaussian process classifier in a meta-learning scheme) in an overlapping patient cohort within the MAC trial (11) and by Ball et al. in an emotion regulation task (37).

To interpret this negative finding regarding diagnostic accuracy for the primary endpoint (HAM-A reduction), it would be desirable to assess the following two questions separately: (1) Does the task fMRI data set convey sufficient information about the diagnostic question of interest? (2) Are the feature extraction and classification methods suitable to derive sufficiently powerful diagnostic models based on that information? In SVM as well as in MVPA in general, these two aspects are highly interconnected (patterns in MVPA are truly multivariate representations and conceptually different from univariate results in standard fMRI group analyses) (64, 76). Therefore, the following observation needs to be interpreted with caution: In *post hoc* univariate whole-brain analyses, we observed a significant main effect of response only for the alternative interoception-based response criterion with the differential contrast (I > E) (Figure S2 in Supplementary Material). This was the feature definition with which the best performance for diagnostic classification was achieved as well (see Exploratory Analyses). Therefore, in this study, the accuracy of SVM models followed the effects seen in univariate analyses. This may indicate that the interoception task did generally not yield sufficient information about the HAM-A based general response (primary outcome). However, one has to keep in mind that multivariate classification models can, in principle, utilize subthreshold information from multiple voxels, but even highly significant univariate group-level results do not guarantee high classification accuracies (2).

Thus, these negative findings may indicate that fear conditioning and extinction (11) as well as emotion regulation (37) may better reflect neural mechanisms involved in CBT and may therefore be more suitable to serve as a predictive tool than an interoceptive task, especially if measures of general anxiety are used as a reference standard for response. The fear conditioning paradigm has also been used to differentiate between PD/AG with and without depressive comorbidity (77).

It is not possible to identify unequivocally the reasons why our diagnostic approach has failed, nonetheless, we believe that it is important to report this negative finding since the methodologically diverse field of diagnostic MVPA in mental disorders is particularly susceptible to publication bias (7, 78, 79). Most importantly, interoceptive accuracy is still considered one of the major factors contributing to the development and maintenance of panic disorder (80).

Compared to other mental disorders (2, 7), few diagnostic MVPA studies have been reported to predict therapy outcomes in PD/AG so far (11, 37). Consequently, only a minority of available methods has been probed in this scenario. No consensus has yet been reached in the field as to which modeling techniques should be preferred (2, 6, 7). This is the first application of whole-brain voxel-based classification in this setting. Voxel-based approaches have been widely used in successful diagnostic modeling in other mental disorders (2, 7) and are commonly used for MVPA of fMRI data beyond diagnostic classification, particularly in combination with SVM (64). SVM are particularly suitable for classification in datasets with a high dimensionality (i.e., number of features) compared to the number of observations (i.e., patients) (81–83). We have applied a combination of techniques for dimensionality reduction (63) with the aim to improve diagnostic performance: (1) imaging data were downsampled to a lower resolution reducing the number of feature by a factor of 1/8 compared to the original preprocessed data, without expected relevant information loss given the original acquisition resolution and data smoothness. (2) Images were masked to exclude voxels outside gray matter. (3) We applied automated FS to only include the 20% most relevant voxels. Literature-based definition of regions of interest (81) was, however, not feasible since no sufficiently reliable prior knowledge for this purpose was available in the literature. We thus selected features based on the dataset itself. Please note that FS was strictly included in the CV to avoid circular reasoning (84). Failure to do that, for example, selection of regions of interest after interpreting univariate group analyses in the same full dataset, is unfortunately a commonly observed mistake leading to overly optimistic estimates of diagnostic accuracies (7). *Post hoc* univariate group effects support the voxel-based approach with automatic FS: effects were only observed in few regions (see *Post Hoc* Univariate Group Comparisons; Figure S2 in Supplementary Material), so that the inclusion of finer-scale within-region patterns, which is possible in voxel-based modeling, seems preferable compared to large-scale inter-regional MVPA.

In addition to inferentially testing these established whole-brain SVM approaches, we explored the influence of methodological choices regarding feature set preparation, modeling parameters, and software. Furthermore, we assessed GPCs as an alternative to SVMs. However, we only observed minor differences in overall diagnostic accuracies and even none of these models reached clinically meaningful diagnostic performance. We thus conclude that the insufficient diagnostic accuracies observed in the hypothesis tests is not caused by insufficient methodological decisions within this range, highlighting the importance of fMRI task choice.

We also explored the diagnostic ability of our multivariate classification approach to predict reduction of symptoms directly related to interoceptive exposure. Better diagnostic accuracies were achieved with this alternative response criterion, particularly with the differential contrast (I > E) combined with simple automated FS (Table 3). Contrasts are used to test specific effects in general linear models of fMRI data (85). The differential contrast is aimed to be more specific for interoception than the simple contrast I as it excludes baseline effects and effects common to both task conditions. Though not directly amenable to statistical testing, results indicate that defining such specific differential contrasts facilitates improved feature extraction compared to simple contrasts (i.e., beta-maps). However, this in an exploratory result without independent validation and still no clinically useful diagnostic accuracies were achieved.

Some limitations apply to this work: subjects were stratified as responders or non-responders. Theoretically, response to therapy can be treated as a regression problem (86) instead of classification, particularly as some non-responders also experienced clinically relevant anxiety reduction. Regression requires larger samples than classification to achieve sufficient statistical power. This is why we favored a classification in this pilot analysis.

The alternative response criterion based on interoception-related symptom reduction relies on self-report data and an IE at home. Thus, there is no external control of symptom relief and patient’s adherence to the task. We can thus not exclude a social desirability bias in the self-report data (87). Results based on this alternative response criterion as well as results of additional methodological comparisons beyond the planned hypothesis tests are exploratory in nature. Generalizability to other samples can thus not be directly concluded.

Data used here were acquired at multiple sites. It is crucial for actual clinical implementations that models generalize to different sites, ideally without a need for site-specific training datasets to address potential between-scanner differences (88, 89). Though MVPA techniques ideally separate informative from uninformative information in the data, it cannot be excluded that site effects may have reduced diagnostic performance in this study.

Models reported in this paper were limited to SVM and limited exploratory assessment of GPC classifiers. Thus, despite similar diagnostic performance of SVM and GPC, results do not generalize to other MVPA algorithms available for analyses of neuroimaging data (2, 6, 7).

## Conclusion

Support vector machine-classification of fMRI data from an interoception task did not prove to be diagnostically applicable to predict individual CBT outcomes in PD/AG as measured by general anxiety reduction. Results contrast with previously reported diagnostic accuracies in models based on alternative tasks with alternative classifiers. Results of an exploratory analysis indicate that the method may be more suitable to predict symptom reduction directly related to an IE and related extent of interoceptive symptom relief. We believe that beyond identifying optimal data analysis strategies, the identification and optimization of suitable paradigms will be an important area of research in developing neuroimaging biomarkers in PD/AG and other anxiety disorders.

## Ethics Statement

Written informed consent was obtained from all participants in accordance with the Declaration of Helsinki. The randomized clinical trial (isrctn.org identifier: ISRCTN80046034) was approved by the ethics committee of the Medical Faculty of the Technische Universität Dresden (agreement EK 164082006). The neuroimaging components were approved by the ethics committee of the Medical Faculty of the RWTH Aachen University, Aachen (agreement EK 073/07) and at all local sites.

## Author Contributions

BS, JB, and BP planned and carried out the analyses and drafted the manuscript. UL, DW, AG, BStr, H-UW, AS, AW, CK, TK, VA, and BP planned and carried out data acquisition for the MAC trial and neuroimaging substudy. They contributed to the interpretation of the results and to revision of the manuscript for important intellectual content. All the authors approved the final version of the manuscript and agreed to be accountable for the content of the work.

## Conflict of Interest Statement

VA is member of the advisory boards and/or gave presentations for the following companies: Astra-Zeneca, Janssen-Organon, Lilly, Lundbeck, Servier, Pfizer, and Wyeth. He chaired the committee for the Wyeth Research Award Depression and Anxiety. TK has received in the past 3 years honoraria or educational grants from Janssen, Bristol Myers-Squibb, Wyeth, Lundbeck, Lilly, Astra-Zeneca, and Pfizer. CK received fees for an educational program from Aristo Pharma, Janssen-Cilag, Lilly, MagVenture, Servier, and Trommsdorff as well as travel support and speakers honoraria from Aristo Pharma, Janssen-Cilag, Lundbeck and Servier. The other authors reported no biomedical financial interests or potential conflicts of interest.

## Footnotes

^1^http://www.fil.ion.ucl.ac.uk/spm.

^2^http://fmri.wfubmc.edu/software/pickatlas.
