# Supplementary material for: Support Vector Machine Analysis of Functional Magnetic Resonance Imaging of Interoception Does Not Reliably Predict Individual Outcomes of Cognitive Behavioral Therapy in Panic Disorder with Agoraphobia
Source: Front Psychiatry. 2017 Jun 9;8:99. doi: 10.3389/fpsyt.2017.00099 (PMC5465291; doi:10.3389/fpsyt.2017.00099)
Supplement: Supplementary file 1 [file Presentation_1.PDF]

Supplementary Material

Support vector machine analysis of functional MRI of interoception does not reliably predict individual outcomes of cognitive behavioral therapy in panic disorder with agoraphobia

Benedikt Sundermann*, Jens Bode, Ulrike Lueken, Dorte Westphal, Alexander L. Gerlach, Benjamin Straube, Hans-Ulrich Wittchen, Andreas Ströhle, André Wittmann, Carsten Konrad, Tilo Kircher, Volker Arolt, Bettina Pfleiderer

*** Correspondence:** Benedikt Sundermann: benedikt.sundermann@uni-muenster.de

1 Supplementary Figures and Tables

1.1 Supplementary Tables

Supplementary Table 1. Demographical and psychometrical characteristics of responders and non-responders regarding interoception related symptom reduction after an interoceptive exposure.

		Responders	Non-responders	test statistic	df	*p*
Number		26	28			
Sex (female:male)	female	19 (73.1 %)	16 (57.1 %)	$\chi2 = 1.501$	1	*0.221*[c]
	male	7 (26.9 %)	12 (42.9 %)			
Age (years)		37.0 ± 11.5	36.8 ± 11.3	t = -0.093	52	*0.926*[a]
Education	lower secondary	13 (50.0 %)	14 (50.0 %)	$\chi2 = 0.306$	2	*0.858*[c]
	higher secondary	9 (34.6 %)	11 (39.3 %)			
	university	4 (15.4 %)	3 (10.7 %)			
Site (n)	Aachen	0 (0.0 %)	1 (3.6 %)	$\chi2 = 1.272$	3	*0.736*[c]
	Berlin	9 (34.6 %)	8 (28.6 %)			
	Dresden	11 (42.3 %)	11 (39.3 %)			
	Münster	6 (23.1 %)	8 (28.6 %)			
CBT arm	therapist-guided	15 (57.7 %)	14 (50.0 %)	$\chi2 = 0.321$	1	*0.571*[c]
	nonguided	11 (42.3 %)	14 (50.0 %)			
Randomized first fMRI condition (n)	Interoception	13 (50.0 %)	14 (50.0 %)	$\chi2 = 0$	1	*1.000*[c]
	Exteroception	13 (50.0 %)	14 (50.0 %)			
HAM-A	before CBT	24.5 ± 5.6	25.00 ± 5.5	t = 0.358	52	*0.722*[a]
	after CBT	13.0 ± 7.5	13.9 ± 5.7	t = 0.476	52	*0.636*[a]
HAM-A based general CBT response	Responders	14 (53.8 %)	13 (46.4 %)	$\chi2 = 0.297$	1	*0.586*[c]
	Non-responders	12 (46.2 %)	15 (53.6 %)			
BDI-II	before CBT	16.3 ± 9.5	16.4 ± 9.2	t = 0.049	52	*0.961*[a]
	after CBT	9.6 ± 9.8	9.7 ± 6.1	t = 0.029	52	*0.977*[a]
ASI	before CBT	30.8 ± 9.7	29.1 ± 11.5	t = -0.558	52	*0.579*[a]
	after CBT	15.8 ± 10.5	15.9 ± 8.5	t = 0.048	52	*0.962*[a]
CGI	panic symptoms	5 (4-7)	5 (4-7)			*0.782*[b]
	anxiety	4 (1-6)	4 (3-5)			*0.401*[b]
PAS		24.2 ± 9.2	26.8 ± 7.6	t = 1.140	52	*0.259*[a]
TMT (seconds)	A	26.4 ± 9.1	26.3 ± 9.0	t = 0.538	52	*0.963*[a]
	B	55.0 ± 17.9	61.1 ± 18.5	t = 1.225	52	*0.226*[a]
Digit Span Task	total	14.7 ± 2.9	14.8 ± 3.3	t = 0.197	52	*0.910*[a]
Comorbid depression (n)[d]	before CBT	9 (34.6 %)	8 (28.6 %)	$\chi2 = 0.228$	1	*0.633*[c]
	after CBT	5 (19.2 %)	2 (7.1 %)	$\chi2 = 1.746$	1	*0.186*[c]

All test-results without further specification were obtained at the first visit at base line assessment of cognitive behavioral therapy (CBT) and represent 'mean ± SD' or 'median (range)'. * denotes statistical significance ($p < 0.05$). [a] t-test. [b] Mann-Whitney-U-test, [c] χ^2-test. [d] based on BDI-II scores CBT: cognitive behavioral therapy, HAM-A: Hamilton Scale for Anxiety, BDI-II: Beck Depression Inventory II, ASI: Anxiety Sensitivity Index, CGI: Clinical Global Impression, PAS: PD/AG Scale, TMT: trail-making task.

Supplementary Table 2. Results of explorative analyses to predict general CBT response with further methodological variations based on the simple contrast (I).

voxel size	mask	FS	classifier	C	accuracy	sensitivity	specificity
4 x 4 x 4 mm	subcortical HO	—	SVM	0.01	49.2 %	46.7 %	51.7 %
				1	50.9 %	56.7 %	44.8 %
				100	50.9 %	56.7 %	44.8 %
4 x 4 x 4 mm	cortical HO	—	SVM	0.01	44.1 %	40.0 %	48.3 %
				1	44.1 %	40.0 %	48.3 %
				100	44.1 %	40.0 %	48.3 %
4 x 4 x 4 mm	AAL	—	SVM	0.01	39.0 %	36.7 %	41.4 %
				1	39.0 %[a]	36.7 %[a]	41.4 %[a]
				100	39.0 %	36.7 %	41.4 %
4 x 4 x 4 mm	AAL	t-test filter	SVM	0.01	39.0 %	40.0 %	37.9 %
				1	39.0 %[a]	40.0 %[a]	37.9 %[a]
				100	39.0 %	40.0 %	37.9 %
4 x 4 x 4 mm	AAL	RFE-SVM	SVM	0.01	39.0 %	36.7 %	41.4 %
				1	39.0 %[a]	36.7 %[a]	41.4 %[a]
				100	39.0 %	36.7 %	41.4 %
4 x 4 x 4 mm	AAL	SVM weighting	SVM	0.01	37.3 %	36.7 %	37.9 %
				1	37.3 %	36.7 %	37.9 %
				100	37.3 %	36.7 %	37.9 %
4 x 4 x 4 mm	amygdala and parahippocampal gyrus	—	SVM	0.01	47.5 %	53.3 %	41.4 %
				1	47.5 %	50.0 %	44.8 %
				100	47.5 %	50.0 %	44.8 %
4 x 4 x 4 mm	subcortical HO	t-test filter	SVM	0.01	49.2 %	53.3 %	44.8 %
				1	49.2 %	53.3 %	44.8 %
				100	49.2 %	53.3 %	44.8 %
4 x 4 x 4 mm	subcortical HO	RFE-SVM	SVM	0.01	52.5 %	60.0 %	44.8 %
				1	52.5 %	60.0 %	44.8 %
				100	52.5 %	60.0 %	44.8 %
4 x 4 x 4 mm	AAL	—	SVM	0.01	39.0 %[b]	36.7 %[b]	41.4 %[b]
				1	39.0 %[b]	36.7 %[b]	41.4 %[b]
				100	39.0 %[b]	36.7 %[b]	41.4 %[b]
2 x 2 x 2 mm	AAL	—	SVM	0.01	37.3 %	40.0 %	34.5 %
				1	37.3 %	40.0 %	34.5 %
				100	37.3 %	40.0 %	34.5 %
2 x 2 x 2 mm	subcortical HO	—	SVM	0.01	49.2 %	50.0 %	48.3 %
				1	49.2 %	53.3 %	44.8 %
				100	49.2 %	53.3 %	44.8 %
6 x 6 x 6 mm	AAL	—	SVM	0.01	35.6 %	36.7 %	34.5 %
				1	35.6 %	36.7 %	34.5 %
				100	35.6 %	36.7 %	34.5 %
6 x 6 x 6 mm	subcortical HO	—	SVM	0.01	49.2 %	53.3 %	44.8 %
				1	39.0 %	43.3 %	34.5 %
				100	39.0 %	43.3 %	34.5 %
4 x 4 x 4 mm	AAL	—	GPC	n/a	39.0 %	36.7 %	41.4 %
4 x 4 x 4 mm	AAL	t-test filter	GPC	n/a	39.0 %	36.7 %	41.4 %

[a] hypothesis test (see main text), [b] tested with the PRoNTo toolbox instead of MANIA. FC: feature selection, C: SVM cost paramerter, HO: Harvard-Oxford atlas, AAL: automated anatomical labeling atlas, RFE-SVM: recursive feature elimination using support vector machines

Supplementary Table 3. Results of explorative analyses to predict general CBT response with further methodological variations based on the differential contrast (I>E).

voxel size	mask	FS	classifier	C	accuracy	sensitivity	specificity
4 x 4 x 4 mm	subcortical HO	—	SVM	0.01	39.0 %	36.7 %	41.4 %
				1	39.0 %	36.7 %	41.4 %
				100	39.0 %	36.7 %	41.4 %
4 x 4 x 4 mm	cortical HO	—	SVM	0.01	37.3 %	33.3 %	41.4 %
				1	37.3 %	33.3 %	41.4 %
				100	37.3 %	33.3 %	41.4 %
4 x 4 x 4 mm	AAL	—	SVM	0.01	39.0 %	30.0 %	48.3 %
				1	39.0 %[a]	30.0 %[a]	48.3 %[a]
				100	39.0 %	30.0 %	48.3 %
4 x 4 x 4 mm	AAL	t-test filter	SVM	0.01	54.2 %	50.0 %	58.6 %
				1	54.2 %[a]	50.0 %[a]	58.6 %[a]
				100	54.2 %	50.0 %	58.6 %
4 x 4 x 4 mm	AAL	RFE-SVM	SVM	0.01	42.4 %	40.0 %	44.8 %
				1	42.4 %[a]	40.0 %[a]	44.8 %[a]
				100	42.4 %	40.0 %	44.8 %
4 x 4 x 4 mm	AAL	SVM weighting	SVM	0.01	44.1 %	40.0 %	48.3 %
				1	44.1 %	40.0 %	48.3 %
				100	44.1 %	40.0 %	48.3 %
4 x 4 x 4 mm	amygdala and parahippocampal gyrus	—	SVM	0.01	50.9 %	56.7 %	44.8 %
				1	42.4 %	46.7 %	37.9 %
				100	42.4 %	46.7 %	37.9 %
4 x 4 x 4 mm	subcortical HO	t-test filter	SVM	0.01	50.9 %	56.7 %	44.8 %
				1	54.2 %	53.3 %	55.2 %
				100	54.2 %	53.3 %	55.2 %
4 x 4 x 4 mm	subcortical HO	RFE-SVM	SVM	0.01	45.8 %	46.7 %	44.8 %
				1	45.8 %	46.7 %	44.8 %
				100	45.8 %	46.7 %	44.8 %
4 x 4 x 4 mm	AAL	—	SVM	0.01	39.0 %[b]	30.0 %[b]	48.3 %[b]
				1	39.0 %[b]	30.0 %[b]	48.3 %[b]
				100	39.0 %[b]	30.0 %[b]	48.3 %[b]
2 x 2 x 2 mm	AAL	—	SVM	0.01	40.7 %	40.0 %	41.4 %
				1	40.7 %	40.0 %	41.4 %
				100	40.7 %	40.0 %	41.4 %
2 x 2 x 2 mm	subcortical HO	—	SVM	0.01	42.4 %	43.3 %	42.4 %
				1	42.4 %	43.3 %	42.4 %
				100	42.4 %	43.3 %	42.4 %
6 x 6 x 6 mm	AAL	—	SVM	0.01	42.4 %	33.3 %	51.7 %
				1	42.4 %	33.3 %	51.7 %
				100	42.4 %	33.3 %	51.7 %
6 x 6 x 6 mm	subcortical HO	—	SVM	0.01	40.7 %	43.3 %	37.9 %
				1	33.9 %	40.0 %	27.6 %
				100	33.9 %	40.0 %	27.6 %
4 x 4 x 4 mm	AAL	—	GPC	n/a	45.8 %	33.3 %	58.6 %
4 x 4 x 4 mm	AAL	t-test filter	GPC	n/a	52.5 %	43.3 $	62.1 %

[a] hypothesis test (see main text), [b] tested with the PRoNTo toolbox instead of MANIA. FC: feature selection, C: SVM cost paramerter, HO: Harvard-Oxford atlas, AAL: automated anatomical labeling atlas, RFE-SVM: recursive feature elimination using support vector machines

Supplementary Figures

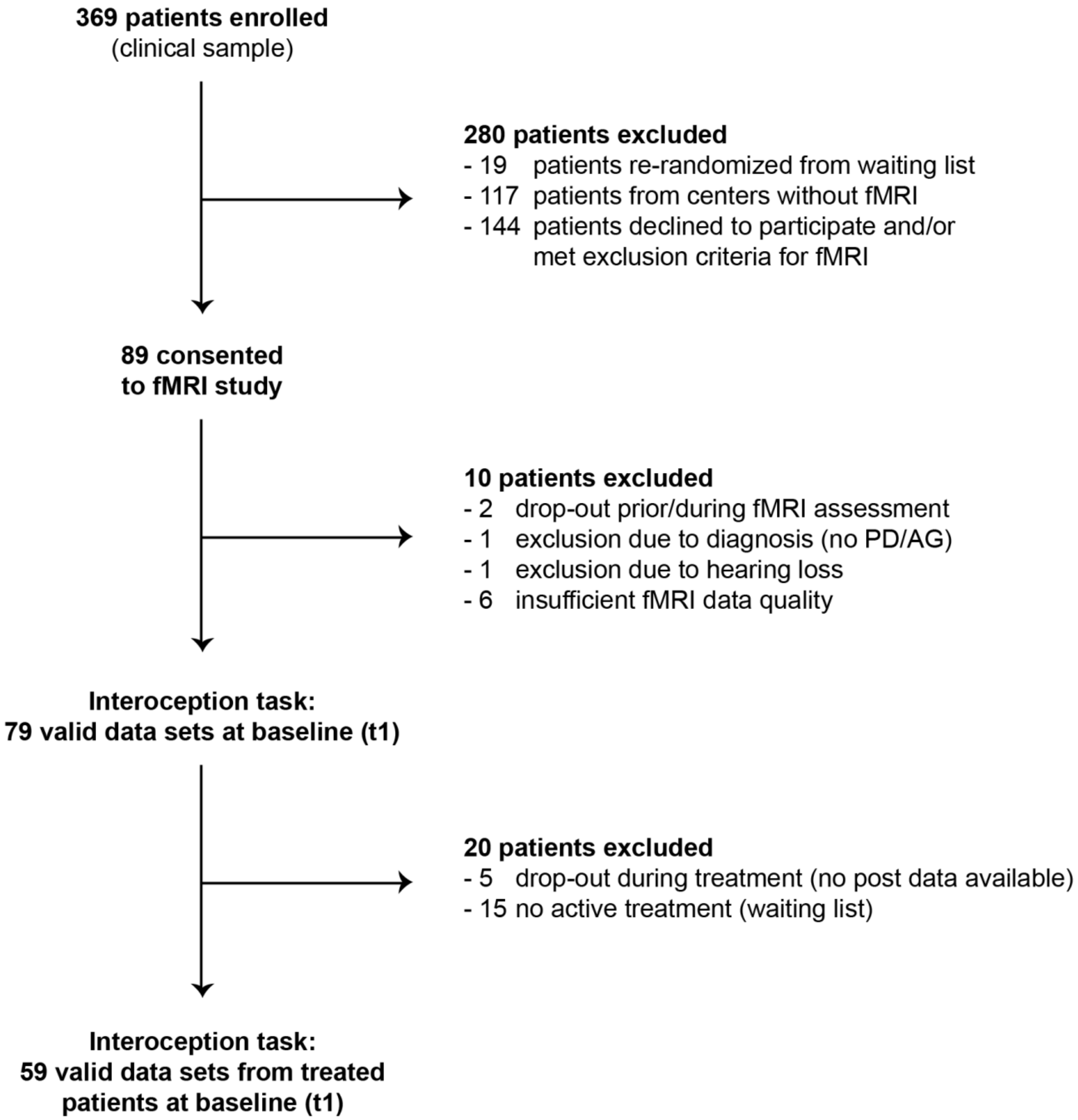

Supplementary Figure 1. Flow-chart of patient selection for diagnostic modelling of functional MRI data from an interoception task.

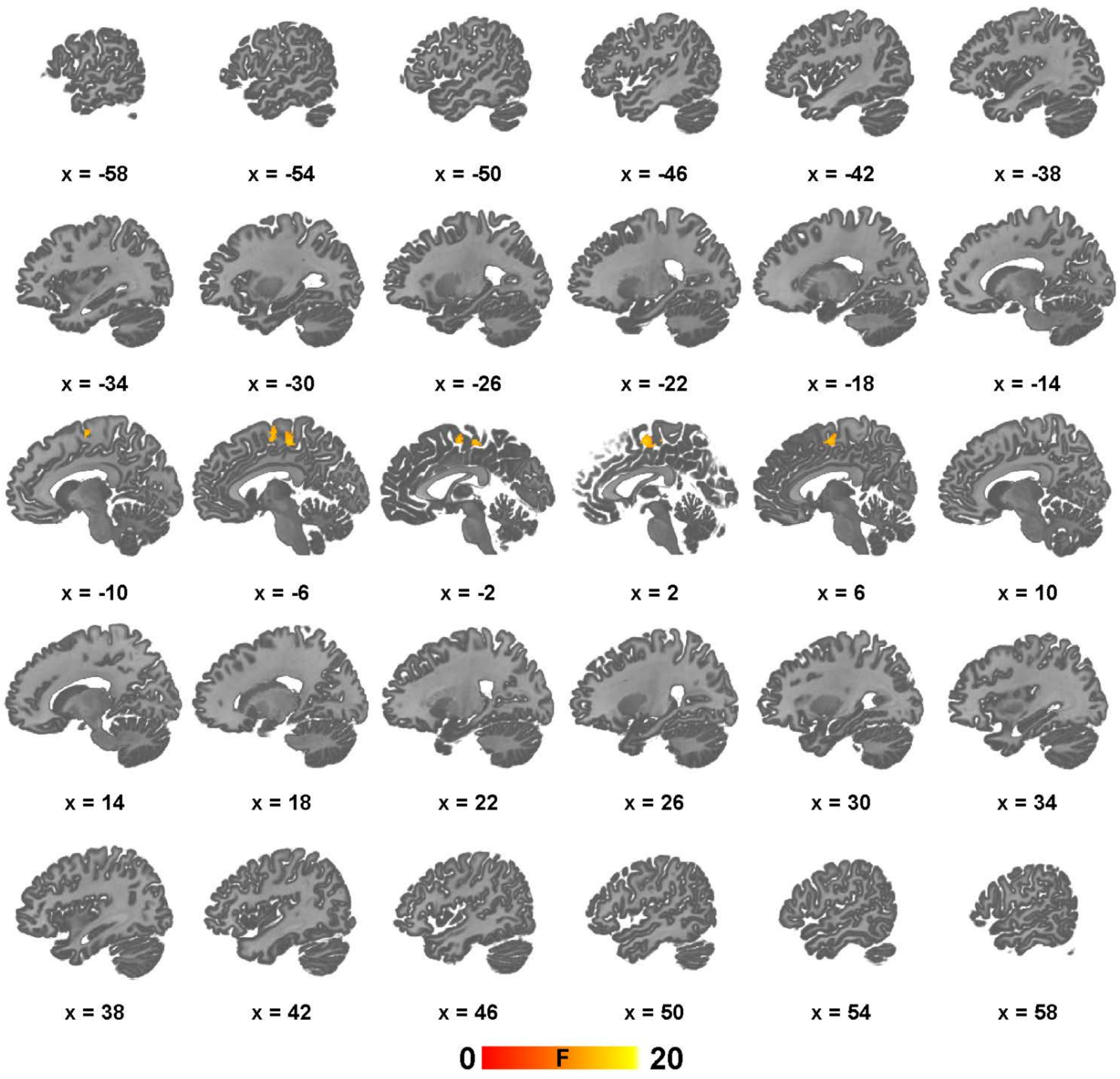

Supplementary Figure 2. Univariate group analysis, main effect of response (response based on interoception criterion), contrast: I > E, cluster-size corrected p < 0.05 (voxel-wise threshold p < 0.001, minimum cluster-size of 42 contiguous voxels)
